# Dye-Enhanced Self-Electrophoretic Propulsion of Light-Driven TiO_2_–Au Janus Micromotors

**DOI:** 10.1007/s40820-017-0133-9

**Published:** 2017-02-18

**Authors:** Yefei Wu, Renfeng Dong, Qilu Zhang, Biye Ren

**Affiliations:** 1 School of Materials Science and Engineering, South China University of Technology, Guangzhou, 510640 People’s Republic of China; 2 School of Chemistry and Environment, South China Normal University, Guangzhou, 510006 People’s Republic of China

**Keywords:** TiO_2_–Au Janus micromotor, Self-electrophoresis, Light-driven, Motion control, Dye pollution, Environmental remediation

## Abstract

Light-driven synthetic micro-/nanomotors have attracted considerable attention in recent years due to their unique performances and potential applications. We herein demonstrate the dye-enhanced self-electrophoretic propulsion of light-driven TiO_2_–Au Janus micromotors in aqueous dye solutions. Compared to the velocities of these micromotors in pure water, 1.7, 1.5, and 1.4 times accelerated motions were observed for them in aqueous solutions of methyl blue (10^−5^ g L^−1^), cresol red (10^−4^ g L^−1^), and methyl orange (10^−4^ g L^−1^), respectively. We determined that the micromotor speed changes depending on the type of dyes, due to variations in their photodegradation rates. In addition, following the deposition of a paramagnetic Ni layer between the Au and TiO_2_ layers, the micromotor can be precisely navigated under an external magnetic field. Such magnetic micromotors not only facilitate the recycling of micromotors, but also allow reusability in the context of dye detection and degradation. In general, such photocatalytic micro-/nanomotors provide considerable potential for the rapid detection and “on-the-fly” degradation of dye pollutants in aqueous environments.

**Electronic supplementary material:**

The online version of this article (doi:10.1007/s40820-017-0133-9) contains supplementary material, which is available to authorized users.

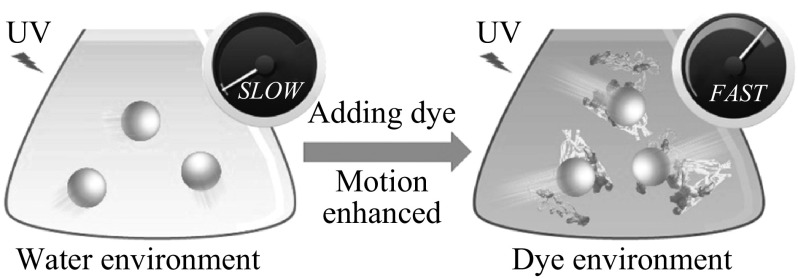

## Highlights

- TiO_2_–Au Janus micromotors can obtain energy from photocatalytic degradation of dyes in aqueous solution and exhibit light-induced dye-enhanced motion through self-electrophoretic effects without additional reagents.
- Micromotors are faster in aqueous dye solutions than in pure water under the same UV light intensity.
- The prepared micromotors are easily synthesized and exhibit excellent reusability in the degradation and detection of dye pollutants.

## Introduction

Nano-/micromotors have received growing attention in recent years because of their potential for application in biomedical and environmental fields [1–14], such as in drug delivery and release [15–18], isolation of biological targets [19], DNA hybridization [20], bacterial detection [21, 22], ion sensing [23], and water treatment [24–29]. Developments in nanotechnology can pave new routes to future practical applications of nano-/micromotors in microscale environments. Among the various nano-/micromotors reported to date, the light-driven micro-/nanomotor based on photocatalytic reactions is particularly attractive due to its unique characteristics, including its remote control, cycling stop-and-go motion, and simple speed control through variation in the light intensity [30–33]. In terms of potential materials for such applications, TiO_2_ is a common, low-cost, and highly efficient photocatalyst capable of decomposing a wide variety of organic and inorganic compounds in both liquid and gaseous states under UV irradiation [34–39]. For example, light-driven micromotors based on TiO_2_ that have been reported to date include plain TiO_2_ particles, TiO_2_ rockets, and TiO_2_-based Janus micromotors. These micromotors are particularly unique in that they can be wirelessly controlled by light, in addition to being able to degrade organic pollutants because of the high photocatalytic activity of TiO_2_. For example, Guan et al. reported that Pt-TiO_2_ Janus micromotors can effectively degrade rhodamine B in water [40]. However, it is currently unclear whether the speed of TiO_2_-based micromotors can be influenced by the different photocatalytic activities of TiO_2_ toward various organic pollutants. In other words, we wish to find out whether the photocatalytic degradation of organic pollutants enhances the self-electrophoretic propulsion of TiO_2_-based Janus micromotors.

**Fig. 1:**
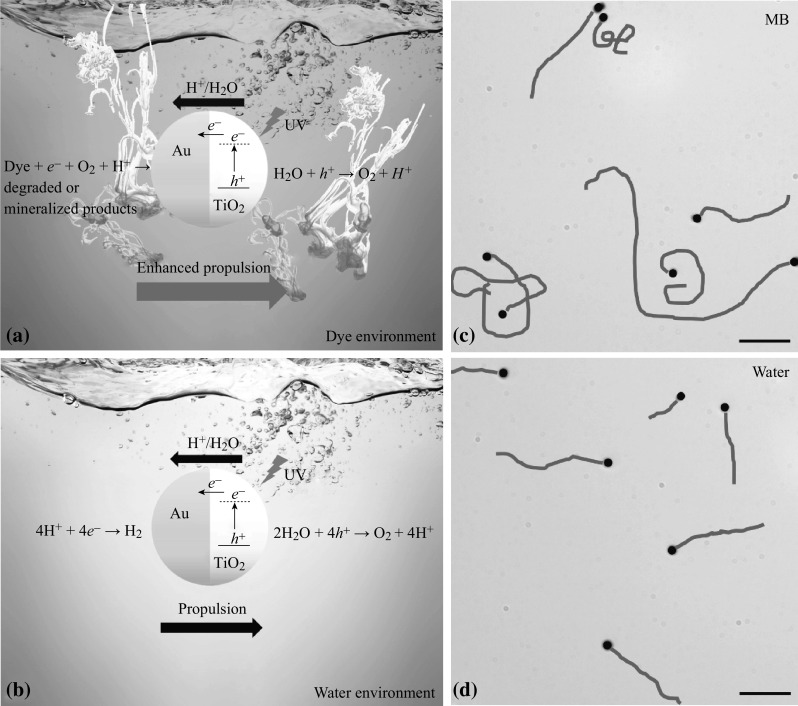
Schematic representation of the propulsion mechanism of the UV light-induced TiO_2_–Au Janus micromotors in: **a** an aqueous dye solution, and **b** pure water. Tracklines of the micromotors in **c** an aqueous solution containing 10^−5^ g L^−1^ MB, and **d** pure water (taken from Video S1), under 40 mW cm^−2^ UV light irradiation over 1 s (*scale bar* 10 μm)

In that context, we herein aim to demonstrate the dye-accelerated motion of light-driven TiO_2_–Au Janus micromotors in a series of aqueous solutions of the dyes methyl blue (MB), cresol red (CR), and methyl orange (MO), as this is a necessary prerequisite for motors employed in environmental applications (Fig. 1a). In general, for light-driven micromotors, strong propulsion requires high luminous energy, which is undesirable for practical applications from an economic standpoint [32]. In contrast, other types of micromotors require high concentrations of chemical fuels (e.g., H_2_O_2_) or additional surfactants (i.e., secondary pollutants) to achieve high speeds [41, 42]. We hope that our findings will demonstrate that the enhanced propulsion of light-driven micromotors is facile under low light energy through the consumption of pollutants present in aqueous environments. We expect that this work will be of great importance for enhancing the efficiency of light-driven Janus micromotors based on photocatalytic reactions and developing green technology for environmental remediation.

## Experimental

### Preparation of the TiO_2_–Au Janus Micromotors

TiO_2_ microspheres were prepared using a previously reported solvent extraction/evaporation method [43]. Firstly, titanium butoxide (1.0 mL, Sigma #244112) was dissolved in ethanol (40 mL), and the resulting solution stirred for 3 min. After this time, the solution was incubated at room temperature for 3 h. The resulting TiO_2_ microspheres were then collected by centrifugation at 8000 rpm for 5 min and washed three times with ethanol (Guangzhou Chemical Reagent Co.) and ultrapure water (18.2 MΩ cm), prior to drying in air at room temperature. The TiO_2_ microspheres were then annealed at 400 °C for 2 h to obtain anatase TiO_2_ microspheres (1.0 μm mean diameter), which were then employed as base particles for the TiO_2_–Au light-driven Janus micromotors. After dispersion of the TiO_2_ particles (1.0 mg) in ethanol (2.0 mL), the resulting suspension was dropped onto glass slides and dried uniformly at ambient temperature to give particle monolayers. These particles were then partially covered with a thin gold layer by 3 cycles of 60 s ion sputtering (Q150T Turbo-pumped ES sputter coater/carbon coater, Quorum). The resulting metal layer thickness is 40 nm, as measured by a Dektak 150 Surface Profiler (Veeco). Finally, the desired TiO_2_–Au Janus micromotors were obtained following sonication of the glass slide in deionized water for 5 s.

### Preparation of the Au–Ni–TiO_2_ Janus Micromotors

For the Au–Ni–TiO_2_ magnetic Janus micromotors, the TiO_2_ particle monolayers were prepared according to the above method. The particles were then sputter-coated with a thin nickel layer over 60 s using a Q150T Turbo-pumped ES sputter coater/carbon coater (Quorum). The nickel layer thickness is 10 nm, as measured by a Dektak 150 Surface Profiler (Veeco). The particles were subsequently sputter-coated with a layer of gold (40 nm) over 3 cycles of 60 s.

### Characterization of the TiO_2_/Au Micromotors

Scanning electron microscopy (SEM) and energy-dispersive X-ray spectroscopy (EDX) were carried out using an EVO 18 Field Emission SEM (Zeiss, Germany). The X-ray diffraction (XRD) patterns of the samples were recorded on an X’Pert Pro X-ray diffractometer (PANalytical, Inc.).

### Velocity Calibration Experiments

Velocity calibration experiments were carried out as follows. A sample (2 μL) of the aqueous suspension containing the TiO_2_–Au Janus micromotors was dropped onto a glass slide. The aqueous dye solution (2 μL) was then dropped onto the slide to allow direct mixing with the micromotor droplets. The samples were then subjected to UV irradiation (intensity = 40 × 10^−3^ W cm^−2^), which was generated using Mercury lamp sockets and a dichroic mirror (DM-400). The motion of the TiO_2_–Au Janus micromotors under UV radiation was observed and recorded at room temperature using an optical microscope (Eclipse Ti–S, Nikon Instrument, Inc.), equipped with 40× objectives, and a Zyla sCMOS digital camera (Andor) using the NIS-Elements AR software (version 4.3). The velocity of the nanoparticles was obtained using the Video Spot Tracker program, which calculates the velocity of the nanoparticles from videos recorded by the microscope system. The velocity of the micromotors was calculated from >50 objects from which an average was taken, and the errors were calculated using Microsoft Excel.

### Electrochemical Potential Measurements

A Tafel plot was used to obtain the potentials established at different segments of the various Janus micromotors (Au and TiO_2_) under UV illumination (Intensity = 0.5 × 10^−3^ W cm^−2^, *λ* = 330–380 nm) in pure water and in aqueous solutions of MB (10^−5^ g L^−1^), CR (10^−4^ g L^−1^), and MO (10^−4^ g L^−1^). Gold and TiO_2_ films (all thicknesses = 100 nm) on ITO glass disks (diameter = 1.0 cm) were used as the working electrodes in the electrochemical potential measurements. A CHI600C electrochemical analyzer/workstation (CH Instruments, Inc.) was employed to measure the potentials at a scan rate of 5 mV s^−1^ over a potential range of −0.2 to 0.3 V.

### Photocatalytic Degradation of the Dyes

To examine the photocatalytic degradation of various dye compounds by the micromotors under UV light irradiation, aqueous suspensions containing 2 × 10^7^ TiO_2_–Au micromotors (200 μL) were added to 20 mL glass bottles containing the different dye compounds (15 mL, 25 μM). Prior to each photoreaction, the various mixtures were stirred in the dark for 30 min to achieve an adsorption–desorption equilibrium for the dye and the dissolved oxygen species on the TiO_2_ surface. After this time, the various mixtures were irradiated by UV light (40 W cm^−2^). The glass bottles were then divided into several groups. At the desired time intervals, samples (3.5 mL) of the suspensions were extracted from the glass bottles and were subjected to centrifugation and filtration. The absorbance of any residual dye in the solution was measured by UV–Vis spectrophotometry (Hitachi U-3010). The degradation rate of the dye was then calculated based on the determined absorbance.

### Calibration of Micromotor Quantities

The number of micromotors present in any given sample was estimated following a process similar to that employed for cell counting using the optical microscope [44]. Firstly, an image of an aqueous sample droplet (0.5 μL) was captured from the 1 mL micromotor solution and was placed on the surface of a glass slide. The total area of the circular drop was estimated by measuring its diameter, and the number of micromotors in a 1/80 portion of the drop was counted. This number was then extrapolated to the whole drop and to the full 200 μL sample to give a total of 2 × 10^7^ micromotors (200 μL of the micromotor solution was added to 15 mL of the contaminated sample).

### Reusability Experiments

For the cycling velocity test, the glass slides containing the Au–Ni–TiO_2_ micromotors were sonicated in deionized water for 5 s. The resulting aqueous solution containing the micromotors was then collected, and the micromotors were separated using an external magnet. The transparent upper solution was discarded, and the micromotors were washed three times with ethanol (Guangzhou Chemical Reagent Co.) and ultrapure water (18.2 MΩ cm). The velocity of the micromotors was then measured repeatedly over three cycles.

For the repeated photodegradation experiments, the Au–Ni–TiO_2_ micromotors were first separated from the above aqueous solution (obtained following sonication) using an external magnet. The transparent upper solution was discarded and replaced with solutions of the three dyes of interest (15 mL, 25 μM). The collected micromotors were then redispersed into the solution by sonication and reused for the photodegradation experiments.

### Quantitative Study on Micromotor Performance

The effect of micromotor quantity (or number) on the photodegradation efficiency was performed by the addition of different quantities (i.e., 50–200 μL, corresponding to 0.5 × 10^7^–2 × 10^7^ micromotors) of the TiO_2_–Au micromotors to 20 mL glass bottles containing 15 mL of each dye solution (25 μM). The following procedure employed the steps previously described for the photocatalytic degradation of dyes.

The influence of UV light intensity on the velocity of the Janus micromotors in aqueous dye solutions was examined using light intensities of 5–40 mW cm^−2^ (due to equipment limits, only ND filters (8×, 16×) could be used to control the intensity). Subsequent steps were as described above for the velocity calibration experiments.

## Results and Discussion

### Janus Structure of the TiO_2_/Au Micromotors

**Fig. 2:**
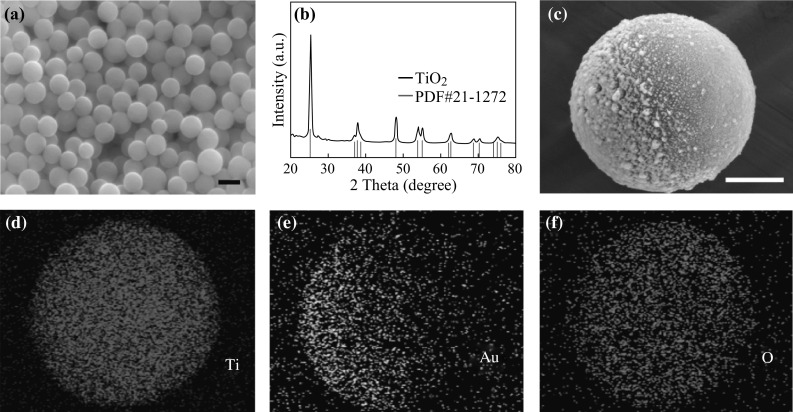
**a** SEM image of the TiO_2_ spheres (*scale bar* 1 μm). **b** XRD pattern of the TiO_2_ spheres. **c** SEM image of a single typical TiO_2_–Au Janus micromotor (*scale bar* 500 nm). **d**–**f** EDX spectroscopy images illustrating the distribution of titanium, gold, and oxygen, respectively, in the micromotors

We initially prepared the TiO_2_–Au Janus micromotors via a previously reported method [41]. The SEM image of the fabricated TiO_2_ spheres on the substrate (Fig. 2a) indicates that they have an average size of 1 μm. In addition, the XRD pattern shown in Fig. 2b suggests that the TiO_2_ particles in the TiO_2_/Au micromotors exhibit an ordered crystalline anatase phase (PDF No. 21-1272). Following the asymmetrical coating of the catalytic TiO_2_/Au Janus micromotors with a thin film (40 nm) of gold on the exposed surfaces of the TiO_2_ particles on a glass slide substrate via an ion sputtering process, analysis by SEM (see Fig. 2c) suggests that the TiO_2_/Au Janus micromotors have an average size of 1 μm. Furthermore, elemental mapping EDX analysis (Fig. 2d–f) of a typical TiO_2_/Au micromotor indicates that Au is asymmetrically distributed on the TiO_2_ particle surfaces, thus confirming the Janus structure of the TiO_2_/Au micromotors.

### Motion of the TiO_2_–Au Janus Micromotors in Aqueous Dye Solutions

**Fig. 3:**
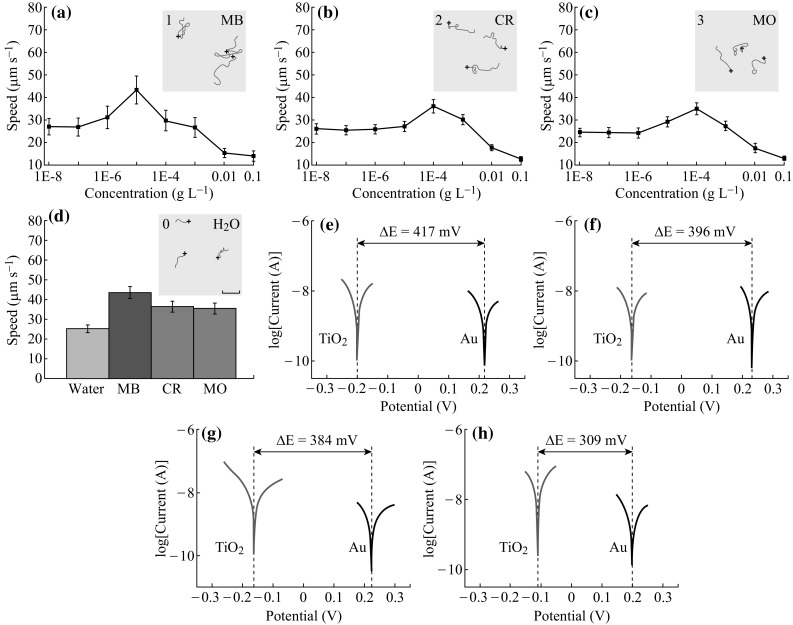
Average velocities of the TiO_2_–Au micromotors at a range of dye concentrations: **a** MB, **b** CR, and **c** MO under 40 mW cm^−2^ UV light irradiation. **d** Average velocities of the TiO_2_–Au micromotors in (*0*) water (*green bar*), (*1*) 10^−5^ g L^−1^ MB (*blue bar*), (*2*) 10^−4^ g L^−1^ CR (*red bar*), and (*3*) 10^−4^ g L^−1^ MO (*pink bar*) under 40 mW cm^−2^ UV light irradiation (*scale bar* 50 μm). Tafel plots of Au (*black lines*) and TiO_2_ (*red lines*) under 40 mW cm^−2^ UV light irradiation in: **e** 10^−5^ g L^−1^ MB, **f** 10^−4^ g L^−1^ CR, **g** 10^−4^ g L^−1^ MO, and **h** pure water. (Color figure online)

We then investigated the motion of the prepared Janus micromotors in an aqueous solution of MB. Interestingly, the TiO_2_–Au Janus micromotors displayed dramatically enhanced propulsion in the MB solution, with a maximum speed of 43.41 μm s^−1^ being calculated from videos recorded by the microscope system in a 10^−5^ g L^−1^ solution of MB under 40 mW cm^−2^ UV light irradiation (Fig. 3a, Video S1), which is 1.7 times faster than the maximum speed recorded in pure water (i.e., 25 μm s^−1^, see Fig. 3d) [30]. Figure 1c, d illustrates the tracklines of the micromotors in the aqueous MB solution and in pure water, respectively, following UV light irradiation (40 mW cm^−2^) for 1 s. It is clear from these figures that the dramatically accelerated motion of the TiO_2_–Au Janus micromotors is caused by activation by the dyes, while the propulsion of the micromotors in pure water originates from light-induced self-electrophoresis [32, 45, 46]. Under UV light, charge separation occurs within the TiO_2_ particles, and electrons are injected from the TiO_2_ conduction band into the Au hemisphere. Protons are then produced from the oxidation of water at TiO_2_, and the resulting electrons are consumed during the reduction of protons at Au. The resulting flux of H^+^ ions generates a fluid flow in the direction of the Au hemisphere, generating a slip velocity and propelling the micromotors. The enhanced propulsion of TiO_2_–Au micromotors in the dye solution involves a similar mechanism to that of self-electrophoresis in pure water (Fig. 1a, b). In this case, the self-electrophoresis is generated by the photocatalytic degradation of the dyes on the asymmetrical surface. In our system, the valence band electrons of TiO_2_ are excited to the conduction band under UV light irradiation at 330–380 nm [47, 48], which facilitates electron transfer from TiO_2_ to Au, suppressing the recombination of electron–hole pairs and enhancing their lifetime [49]. The separated electron–hole pairs then promote the efficient formation of reactive superoxide radicals, which react with the dye molecules to induce their oxidative degradation, yielding degraded or mineralized products [50–53]. Equations (1) and (2) below summarize the main reactions taking place during the photocatalytic degradation of the dyes:

1 $${\text{h}}^{ + } + {\text{H}}_{2} {\text{O}} \to {\text{O}}_{2} + {\text{H}}^{ + }$$

2 $${\text{Dye}} + {\text{O}}_{2} + {\text{e}}^{ - } + {\text{H}}^{ + } \to {\text{degraded or mineralized products}}$$

As per the above equations, H^+^ is highly concentrated on the TiO_2_ side, and a local electric field pointing from the TiO_2_ end to the Au end is formed [54]. The TiO_2_–Au Janus micromotors could therefore be driven by the local electric field with the TiO_2_ side pointing forward through electrophoresis. Indeed, we clearly observe that the TiO_2_–Au Janus micromotors move toward the TiO_2_ side in a 10^−5^ g L^−1^ aqueous solution of MB under UV irradiation (Video S2).

To further confirm this interesting phenomenon, we systematically investigated micromotor motion in aqueous solutions of CR and MO, and the relationship between the TiO_2_–Au Janus micromotor velocity and the dye concentration is shown in Fig. 3 (see also Video S3-5). As expected, micromotor motion is significantly enhanced in the presence of both dye molecules. Indeed, Fig. 3d shows that under the same UV light intensity, the maximum speeds of the micromotors in 10^−4^ g L^−1^ CR and 10^−4^ g L^−1^ MO solutions are approximately 1.5 and 1.4 times faster, respectively, than that recorded in pure water (Video S1). More specifically, the trajectories of the TiO_2_–Au micromotors in the MB (Fig. 3d1), CR (Fig. 3d2), and MO (Fig. 3d3) solutions in addition to that obtained in pure water (Fig. 3d0) under UV irradiation for 5 s reflect the corresponding maximum velocities. We also investigated the motion of pure TiO_2_ spheres in the aqueous dye solutions (Fig. S1) and found that the spheres remained essentially motionless. In contrast, the Au–TiO_2_ micromotors moved more rapidly than the TiO_2_ spheres, although a decrease in Au–TiO_2_ speed was observed upon increasing the dye concentration over a critical concentration. This can be attributed to Ohm’s law. More specifically, the presence of additional ions upon the introduction of the dye species results in an increase in the solution conductivity with increasing dye concentration, which in turn results in the ions weakening self-electrophoresis through a decrease in the inner electric field with increasing solution conductivity [55]. Indeed, it is clear from Fig. 3a–c that the self-electrophoresis mechanism plays a key role in micromotor motion at low dye concentrations and where self-electrophoresis is essentially not affected by the presence of low ion concentrations. As the dye concentration increases, ion effects will become dominant, thus leading to a decrease in micromotor velocity. This can be summarized by the micromotors exhibiting a peak velocity through a balance of the positive self-electrophoresis effect and the negative ion effect in dye-containing solutions.

**Fig. 4:**
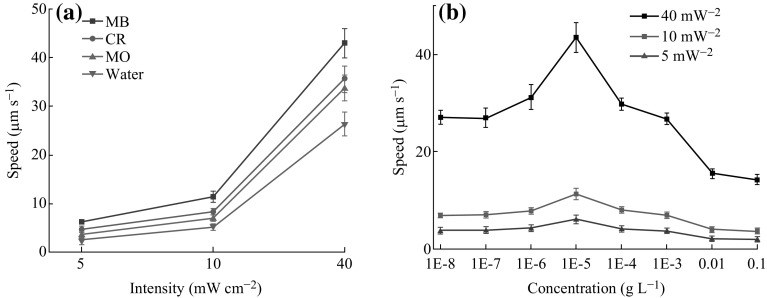
**a** Influence of UV light intensity on the maximum velocities of the Janus micromotors. **b** Influence of UV light intensity on the velocities of Janus micromotors in aqueous solutions containing different MB concentrations

Furthermore, for common light-driven micromotors, the propulsion can be enhanced by increasing the light intensity, *I* [32]. In this case, Fig. 4a shows that by increasing *I* from 5 to 40 mW cm^−2^, the maximum velocity of the micromotors increases from 6.74 to 43.41 μm s^−1^ in 10^−5^ g L^−1^ MB, from 4.98 to 36.31 μm s^−1^ in 10^−4^ g L^−1^ CR, from 4.81 to 35.15 μm s^−1^ in 10^−4^ g L^−1^ MO, and from 2.51 to 26.53 μm s^−1^ in pure water. Moreover, Fig. 4b shows the micromotor speeds at range of MB concentrations (i.e., from 10^−8^ to 10^−1^ g L^−1^) under different light intensities. As shown, the maximum speed is achieved consistently in 10^−5^ g L^−1^ aqueous solution of MB at all UV light intensities examined. Similar observations were made for the CR and MO solutions.

### Electrochemical Potential Measurements

According to previous reports, self-electrophoretic propulsion can be attributed to the mixed potential difference between the two electrodes [46]. For enhanced propulsion in such light-driven micromotors, we examined the mixed potentials between the Au and TiO_2_ electrodes in solutions of MB (10^−5^ g L^−1^), CR (10^−4^ g L^−1^), and MO (10^−4^ g L^−1^) and in pure water under UV light irradiation (Fig. 3e–h). As shown in Fig. 3h, the lowest potential difference (Δ*E* = 309 mV) between the Au and TiO_2_ electrodes is observed in pure water, while the potential differences between the two electrodes in the MB, CR, and MO solutions are 417, 396, and 384 mV, respectively. The larger potential difference between the Au and TiO_2_ electrodes in all three dye solutions compared to that in pure water clearly reflects the enhanced propulsion of the TiO_2_–Au Janus micromotors. In addition, the decrease in potential differences in the order Δ*E*_MB_ > Δ*E*_CR_ > Δ*E*_MO_ is consistent with the observed speeds of the enhanced motion in individual dye solutions (i.e., *V*_MB_ > *V*_CR_ > *V*_MO_). We also examined the mixed potentials between the Au and TiO_2_ electrodes in 10^−1^–10^−8^ g L^−1^ aqueous solutions of MB (Fig. S2), and Tafel plots indicate that the variation in the mixed potentials between the electrodes is consistent with the velocities of the TiO_2_–Au Janus micromotors in different dye concentrations (Fig. 3). This further confirms that the different velocities observed in solution can be attributed to differences in electrochemical potentials. Analogous results were observed for aqueous CR and MO solutions.

### Photocatalytic Degradation of Dyes

**Fig. 5:**
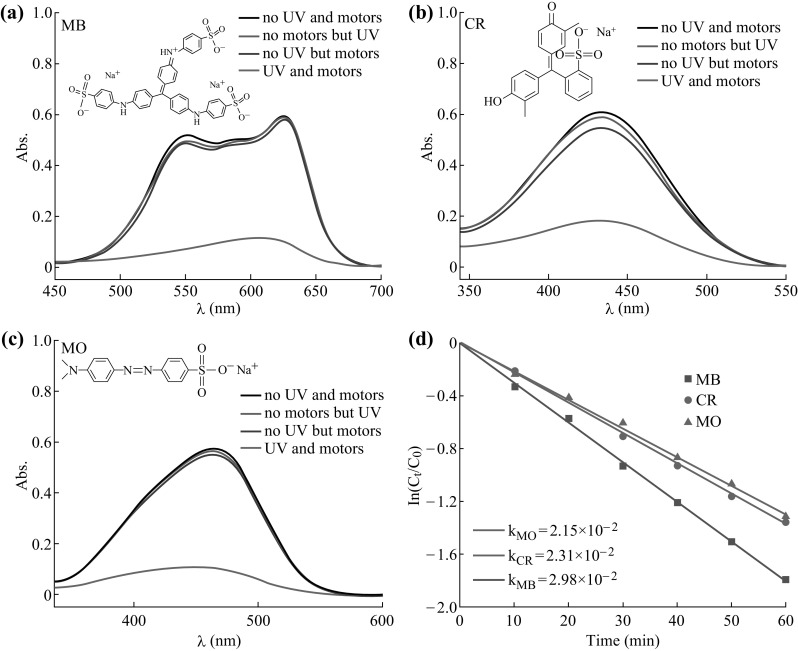
UV–Vis absorbance spectra of aqueous solutions of the three dyes (MB, CR, and MO, 25 μM) following treatment under a range of conditions over 60 min: No UV light or micromotors (*black line*); no micromotors but UV light (*red line*); no UV light but micromotors (*blue line*); UV light and micromotors (*pink line*). **a** MB, **b** CR, **c** MO, and **d** Photodegradation rates of MB, CR, and MO in aqueous solutions containing TiO_2_–Au Janus micromotors under UV irradiation. (Color figure online)

Considering the greatly enhanced electrophoretic propulsions of micromotors in different dye environments, it is important to systematically demonstrate the relationship between the micromotor velocity and the photodegradation rate of the three dyes (MB, CR, and MO) using a series of control experiments. More specifically, dye degradation can be evaluated through comparison of the decreasing absorption intensity of the solution with the absorption intensity of standard solutions through UV–Vis absorption spectroscopy (Fig. S3). The spectra of the three dyes in aqueous solution following treatment under a range of conditions over 60 min are shown in Fig. 5. As shown in Fig. 5a–c, the absorbance of the dye solution containing TiO_2_–Au Janus micromotors decreases following UV irradiation for 60 min, which is indicative of the significant degradation of these dyes. More specifically, the decomposition rates of these dyes (as determined from their standard curves) are 83.3%, 74.1%, and 73.2% for MB, CR, and MO, respectively. The results are consistent with the speeds of the micromotors in the three dye solutions. In contrast, the absorbance of the dye solution remains relatively constant in the other control experiments, and as such, it is clear that dye degradation results from the combined effect of both UV light and TiO_2_–Au micromotors. Subsequently, we further evaluated the photocatalytic degradation rates of MB, CR, and MO in aqueous solutions containing TiO_2_–Au Janus micromotors under UV light. As shown in Fig. 5d, the degradation of all three dyes (at initial concentrations of 25 μM) follows the first-order kinetics model (Eq. 3),

3 $$\ln \frac{{C_{t} }}{{C_{0} }} = kt$$

where *C*_0_ and *C*_*t*_ are the initial concentration and the concentration at time *t*, respectively, and *k* is the first-order rate constant. Figure 5d shows the effect of TiO_2_–Au Janus micromotors on the photodegradation rates of MB, CR, and MO under UV light. As shown, the *k* values decrease in the order *K*_MB_ = 2.98 × 10^−2^ > *K*_CR_ = 2.31 × 10^−2^ > *K*_MO_ = 2.15 × 10^−2^ min^−1^, which corresponds well with the speeds of the TiO_2_-based micromotors in each dye solution. It is therefore clear that the energy required to enhance micromotor propulsion originates from dye degradation, with the decomposition rates indicating that the speed strongly depends on the type of dye.

**Fig. 6:**
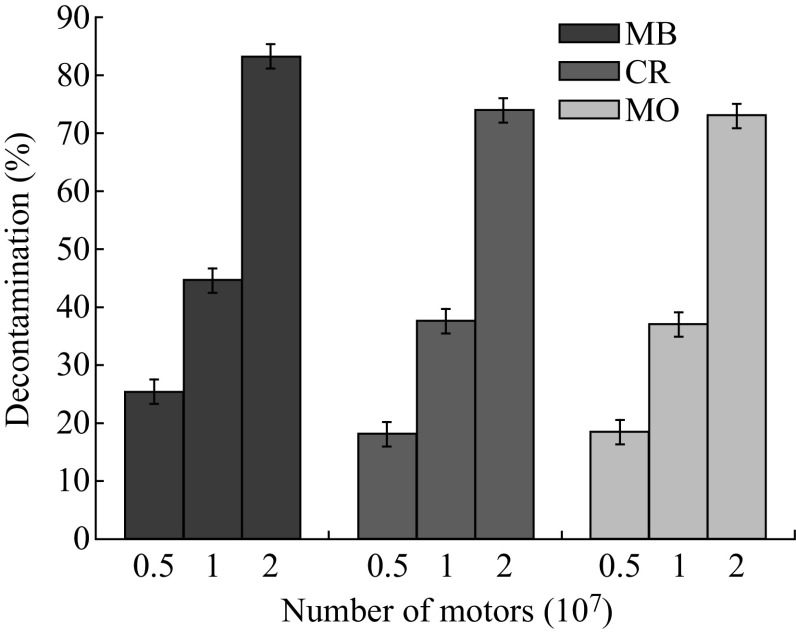
Effect of the number of micromotors on the photodegradation efficiency of MB (*blue bar*), CR (*red bar*), and MO (*orange bar*) under UV light irradiation over 60 min. (Color figure online)

Moreover, as expected, highly concentrated micromotors increase the photodegradation rates of the dyes. As shown in Fig. 6, the quantity of micromotors employed has a significant impact on the degradation efficiency, with the extent of degradation increasing from 25.4% to 83.3% for MB, 18.2% to 74.1% for CR, and 18.6% to 73.2% for MO when the number of micromotors is increased from 0.5 × 10^7^ to 2 × 10^7^.

### Direction Control and Reusability of the Micromotors

**Fig. 7:**
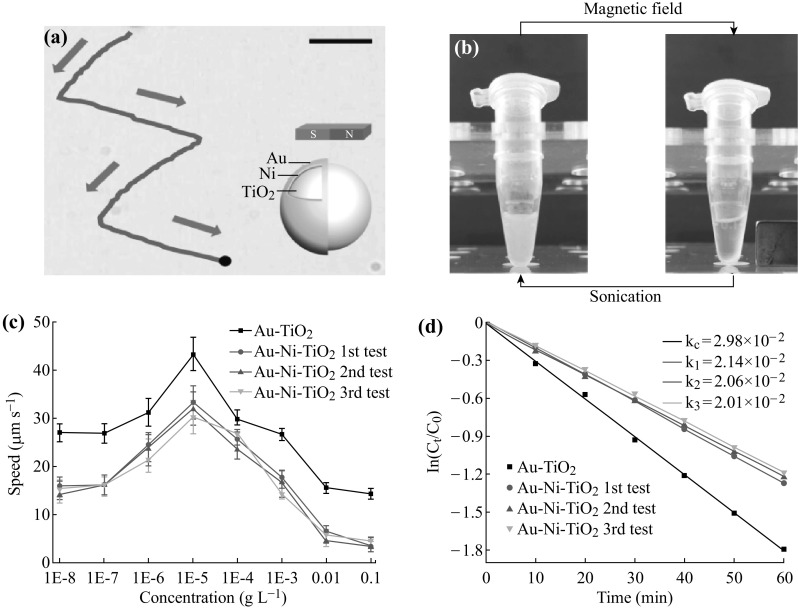
**a** Time-lapse images (taken from Video S6) of the magnetically guided propulsion of Au–Ni–TiO_2_ micromotors under 40 mW cm^−2^ UV light irradiation (*scale bar* 10 μm). **b** Magnetic separation and redispersion process for the Au − Ni − TiO_2_ micromotors. Conditions: 2 μL micromotors dispersed in 100 μL aqueous solution and collected using a Neodymium magnet (power Level N52) over 30 s. **c** Average velocities of the Au–TiO_2_ Janus micromotors (*black line*), and Au–Ni–TiO_2_ micromotors after recycling once (*red line*), twice (*blue line*), and three times (*pink line*) at different MB concentrations under 40 mW cm^−2^ UV light irradiation. **d** Photodegradation rate of MB in a solution containing Au–TiO_2_ Janus micromotors (*black line*), and Au–Ni–TiO_2_ micromotors after recycling once (*red line*), twice (*blue line*), and three times (*pink line*) under UV light irradiation. Reaction time: 60 min. (Color figure online)

The direction control and reusability of micromotors are two key factors when considering the potential practical applications of these materials. In this context, a paramagnetic Ni layer was deposited between the Au layer and TiO_2_ to achieve magnetic control of the directionality of the light-driven TiO_2_–Au Janus micromotors and to allow the micromotors to be recycled [17, 32]. The structure of these magnetic guided Janus micromotors is illustrated in Fig. 7a. Upon the application of an external magnetic field, the micromotors can be precisely navigated along predetermined trajectories (Fig. 7a, Video S6) and can also be recycled (Fig. 7b). However, such magnetic micromotors exhibit slightly lower velocities and weaker photocatalytic activities than the corresponding Au–TiO_2_ micromotors (Fig. 7c, d), likely due to the weaker propulsion and reduced electron–hole separation, as illustrated by the effect of the Ni layer on the potential differences [27, 46]. However, although the recyclable micromotors exhibit reduced propulsion and photocatalytic performance, they have good motion repetition and photodegradation stability for organic pollutants. As shown in Fig. 7c, the maximum velocities of the reused Au–Ni–TiO_2_ micromotors are 33.41, 32.11, and 30.64 μm s^−1^ in a 10^−5^ g L^−1^ solution of MB over 3 repeated tests (Video S7). These values are comparable to the maximum velocity of the Au–TiO_2_ micromotors in the same solution. Moreover, the photodegradation rates of the MB dye in a solution containing the magnetic micromotors are 0.0214, 0.0206, and 0.0201 over 3 repeated measurements (Fig. 7d), indicating that such magnetic direction-controlled and recyclable micromotors have great potential for practical application in environmental remediation. It should also be noted that both the Au–TiO_2_ and the Au–Ni–TiO_2_ micromotors can be recycled via a centrifugation method, with both materials exhibiting stable reusability.

## Conclusions

In conclusion, we observed that TiO_2_–Au Janus micromotors can obtain energy from the photocatalytic degradation of dyes in aqueous solutions without the requirement for any additional reagents. These micromotors also exhibit light-induced dye-enhanced motion through self-electrophoretic effects in dye solutions under UV irradiation. The velocities of the motors in 10^−5^ g L^−1^ MB, 10^−4^ g L^−1^ CR, and 10^−4^ g L^−1^ MO solutions are approximately 1.7, 1.5, and 1.4 times faster, respectively, than those observed in pure water under the same UV light intensity. In addition, we found that the micromotor velocity strongly depends on the type of dyes employed, due to their different photodegradation rates. Furthermore, these micromotors exhibit excellent reusability in the degradation and detection of dye pollutants. These findings indicate the potential for tuning the motion of photocatalytic micro-/nanomotors in addition to “on-the-fly” degradation of dye pollutants in aqueous environments.

## Electronic supplementary material

Below is the link to the electronic supplementary material.

Supplementary material 1 (PDF 521 kb)

Supplementary material 2 (AVI 1472 kb)

Supplementary material 3 (AVI 106 kb)

Supplementary material 4 (AVI 9877 kb)

Supplementary material 5 (AVI 9842 kb)

Supplementary material 6 (AVI 10502 kb)

Supplementary material 7 (AVI 1139 kb)

Supplementary material 8 (AVI 3234 kb)
