# Supplementary material for: Dye-Enhanced Self-Electrophoretic Propulsion of Light-Driven TiO2–Au Janus Micromotors
Source: Nanomicro Lett. 2017 Feb 18;9(3):30. doi: 10.1007/s40820-017-0133-9 (PMC6199027; doi:10.1007/s40820-017-0133-9)
Supplement: Supplementary file 1 — Supplementary material 1 (PDF 521 kb) [file 40820_2017_133_MOESM1_ESM.pdf]

Supporting Information for

Dye-enhanced Self-Electrophoretic Propulsion of Light-Driven TiO_2-Au Janus Micromotors

Yefei Wu[1], Renfeng Dong[2,*], Qilu Zhang[1], Biye Ren[1,*]

[1] School of Materials Science and Engineering, South China University of Technology, Guangzhou 510640, People's Republic of China

[2] School of Chemistry and Environment, South China Normal University, Guangzhou 510006, People's Republic of China

*Corresponding authors. E-mail: dongrenfeng@126.com, mcbyren@scut.edu.cn

Tel.: +86-20-87112708

Videos

Video S1 The motion of TiO_2-Au Janus micromotors in 10^{-5} g L^{-1} Methyl Blue (MB), 10^{-4} g L^{-1} Cresol Red (CR), 10^{-4} g L^{-1} Methyl Orange (MO) aqueous solutions and pure water, respectively

Video S2 The moving direction of TiO_2-Au Janus micromotors under UV in MB aqueous solution

Video S3 The motion of TiO_2-Au Janus micromotors in different concentration of MB aqueous solution

Video S4 The motion of TiO_2-Au Janus micromotors in different concentration of CR aqueous solution

Video S5 The motion of TiO_2-Au Janus micromotors in different concentration of MO aqueous solution

Video S6 Directional control of Au-Ni- TiO_2 Janus micromotors in MB aqueous solution

Video S7 The motion of Au-Ni- TiO_2 Janus micromotors in MB aqueous solution

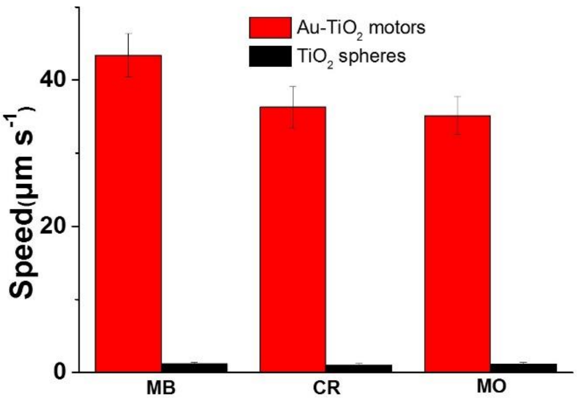

Fig. S1 The motion speeds of Au-TiO_2 motors (red) and pure TiO_2 spheres (black) in 10^{-5} g L^{-1} Methyl Blue (MB), 10^{-4} g L^{-1} Cresol Red (CR), 10^{-4} g L^{-1} Methyl Orange (MO), respectively.

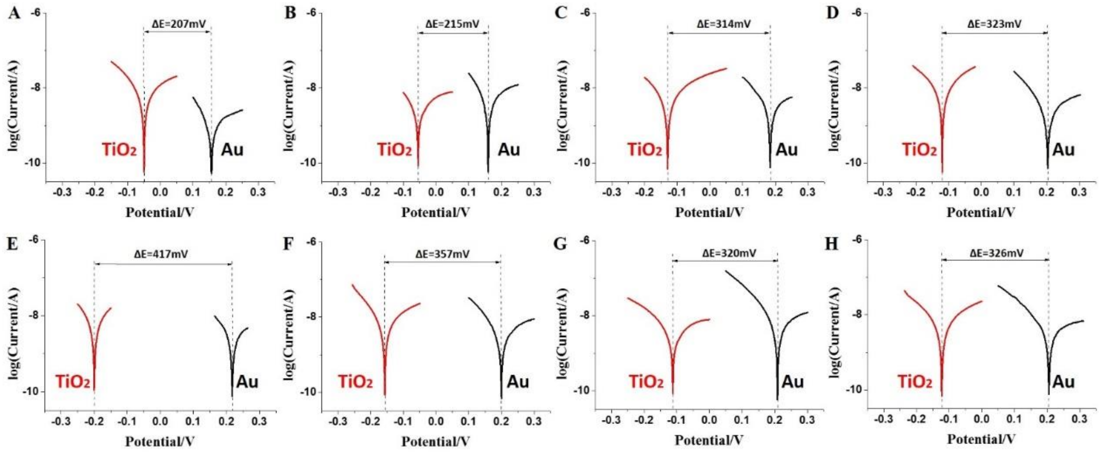

Fig. S2 Tafel plots of Au (black lines) and TiO_2 (red lines) under UV light in (A)-(H) 10^{-1}~10^{-8} g L^{-1} MB aqueous solutions, respectively

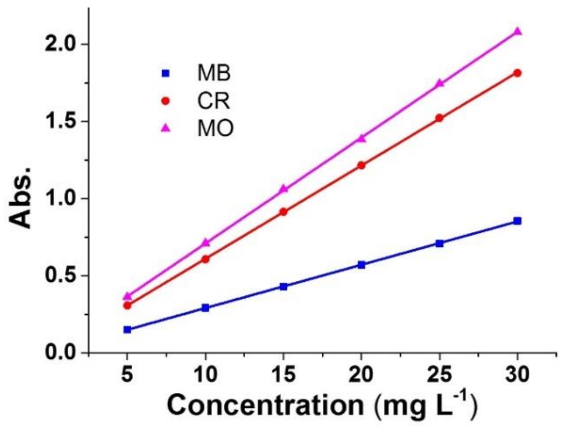

Fig. S3 Absorption–concentration standard curve of MB, CR and MO in aqueous solutions

The absorption intensity *A* of MB, CR, and MO in aqueous solutions were proportional to their concentrations *C*. The working curves were obtained by linearly fitting the data of *A* and *C*. The obtained linear equations are below:

$$A\ (MB) = 0.0281C + 0.0095\ (r^2 = 0.9999) \qquad (1)$$

$$A\ (\mathrm{CR}) = 0.0605C + 0.0049\ (r^2 = 0.9999) \qquad (2)$$

$$A\ (\mathrm{MO}) = 0.0687C + 0.0227\ (r^2 = 0.9998) \qquad (3)$$
